# Burden, associated risk factors and adverse outcomes of gestational diabetes mellitus in twin pregnancies in Al Ain, UAE

**DOI:** 10.1186/s12884-020-03289-w

**Published:** 2020-10-12

**Authors:** Juma Alkaabi, Raya Almazrouei, Taoufik Zoubeidi, Fatema M. Alkaabi, Fatima Rashid Alkendi, Amel Eisa Almiri, Charu Sharma, Abdul-Kader Souid, Nasloon Ali, Luai A. Ahmed

**Affiliations:** 1 Department of Internal Medicine, College of Medicine, and Health Sciences, Emirates University, PO Box 17666, Al Ain, United Arab Emirates; 2 Division of Endocrinology, Tawam Hospital, Al Ain, United Arab Emirates; 3 Departments of Statistics, College of Sciences, United Arab Emirates University, Al Ain, United Arab Emirates; 4 Division of Endocrinology, Al Ain Hospital, Al Ain, United Arab Emirates; 5 Internal Medicine Department, Tawam Hospital, Al Ain, United Arab Emirates; 6 Department of Pediatrics, College of Medicine & Health Sciences, United Arab Emirates University, Al Ain, United Arab Emirates; 7 Institute of Public Health, College of Medicine and Health Sciences, United Arab Emirates University, Al Ain, United Arab Emirates

**Keywords:** Gestational diabetes mellitus, Maternal outcomes, Neonatal outcomes, Twin pregnancy

## Abstract

**Background:**

Gestational diabetes mellitus (GDM) in singleton pregnancies represent a high-risk scenario. The incidence, associated factors and outcomes of GDM in twin pregnancies is not known in the UAE.

**Methods:**

This was five years retrospective analysis of hospital records of twin pregnancies in the city of Al Ain, Abu Dhabi, UAE. Relevant data with regards to the pregnancy, maternal and birth outcomes and incidence of GDM was extracted from two major hospitals in the city. Regression models assessed the relationship between socio-demographic and pregnancy-related variables and GDM, and the associations between GDM and maternal and fetal outcomes at birth.

**Results:**

A total of 404 women and their neonates were part of this study. The study population had a mean age of 30.1 (SD: 5.3), overweight or obese (66.5%) and were majority multiparous (66.6%). High incidence of GDM in twin pregnancies (27.0%). While there were no statistical differences in outcomes of the neonates, GDM mothers were older (OR: 1.09, 95% CI: 1.06–1.4) and heavier (aOR: 1.02, 95% CI: 1.00 -1.04). They were also likely to have had GDM in their previous pregnancies (aOR: 7.37, 95% CI: 2.76–19.73). The prognosis of mothers with twin pregnancies and GDM lead to an independent and increased odds of cesarean section (aOR: 2.34, 95% CI: 1.03–5.30) and hospitalization during pregnancy (aOR: 1.60, 95% CI: 1.16–2.20).

**Conclusion:**

More than a quarter of women with twin pregnancies were diagnosed with GDM. GDM was associated with some adverse pregnancy outcomes but not fetal outcomes in this population. More studies are needed to further investigate these associations and the management of GDM in twin pregnancies.

## Background

The prevalence of gestational diabetes mellitus (GDM) varies worldwide and among racial and ethnic groups, generally in parallel with the prevalence of type 2 diabetes. In addition, the prevalence of GDM also varies because of differences in screening practices, population characteristics, testing method, and diagnostic criteria. The prevalence of GDM has been increasing over time, likely due to increases in mean maternal age and weight, particularly increasing obesity [1–4].

GDM is a major cause for concern during pregnancy. It is associated with several adverse outcomes such as cesarean section, pre-eclampsia, macrosomia, shoulder dystocia, neonatal intensive care unit admission, and perinatal death [5–9]. Moreover, mothers with GDM have increased risk of type 2 diabetes and cardiovascular diseases later in life, while their children have an increased risk for obesity, impaired glucose tolerance and cardiovascular risk profile during adolescence and early adulthood [10, 11]. The greater rate of obesity in women of reproductive age in the current times, pregnancy at later ages and different diagnostic criteria all contribute as many other factors to the increased incidences of GDM in pregnancies [12].

As much GDM has been increasing around the world, so has the rate of twin pregnancy [13]. Concurrent increase in prevalence of GDM and twin pregnancies would ultimately show associations between the two. Previous studies have shown multiple pregnancies to be a risk factor of GDM [14]. However, although twin pregnancies are often associated with high risk of cesarean section, pre-eclampsia, hypoglycemia, retinopathy, preterm birth and low birth weight [15–20], GDM in twin pregnancies have shown conflicting associations to these adverse outcomes [21–24].

Both singleton and twin GDM pregnancies are high-risk pregnancies which require optimal management [25]. In the United Arab Emirates (UAE) the rate of GDM was reported to vary from 7.9% to 24.9% depending on the used diagnostic criteria [26–28], however, one study reported a prevalence of 37.7% when using International Association of Diabetes and Pregnancy Study Group (IADPSG) diagnostic criteria [29]. On the other hand, multiple birth in the UAE is relatively higher [30] than other developed countries [31]. However, currently there is little evidence to guide the management of GDM twin pregnancies as there has been no systematic evaluation of GDM and its burden, effects and causes in twin pregnancies in the UAE. Knowledge of this burden and the associated risk factors and adverse outcomes of GDM is needed to identify possible preventive strategies. Such knowledge will provide direct significant clinical impacts on the management of women with twin pregnancies.

Therefore, the aim of this study is to determine the burden of GDM in twin pregnancies in Al Ain city, UAE, and to explore its associations with adverse maternal and neonatal outcomes.

## Methods

### Study design

This retrospective study on hospital-based data was performed in the two university-affiliated institutions, Tawam and Al Ain Hospital, in Al Ain, Abu Dhabi, UAE (IRB Approval: 308/14). Medical records of all twin pregnancies between 2009 and 2013 were reviewed. A total of 521 twin pregnancies during the study period were identified. Of those, 22 mothers with single pregnancy (miscoded as a twin pregnancy), seven with a pre-existing diabetes, and 88 who delivered at other institutions were excluded. Relevant data (such as socio-demographics, smoking status of the mother, pregnancy related information, labor and delivery details, morbidities and mortalities of both the mother and the twins, hospitalizations during pregnancy and other pregnancy complications) were retrieved from the medical records for the purpose of the study. Major malformations were documented regardless of severity. Postnatal death was set as an event that occurred in the first six months of life. Respiratory distress syndrome was diagnosed based on the clinical picture. Premature birth was defined as birth before 37 weeks of pregnancy are completed. Fetal macrosomia was defined as birthweight above the 90^th^ percentile for gestational age, however it was not weight corrected for twins and not population specific.

All pregnancies in our institutions are screened for GDM between 24 and 28 weeks of gestation. Diagnosis of GDM is made if: fasting plasma glucose > 5.1 mmol/l; or one-hour plasma glucose > 10.0 mmol/l; or two-hour plasma glucose ≥ 8.5 mmol/l. Women with confirmed GDM are offered advice and education on self-monitoring, ideal levels of control with target capillary glucose concentration of pre-prandial ≤ 5.3 mmol/L and either 1-h post meal ≤ 7.8 mmol/L or 2-h post meal ≤ 6.7 mmol/L, risks to current and to subsequent pregnancies. Information on treatment practices for women with GDM were also collected. The study was approved by the Ethics Committee of the College of Medicine and Health Sciences at the United Arab Emirates University (Approval Reference: CRD308/14).

### Statistical analysis

Descriptive statistics were performed to show and compare the distribution of characteristics of the study population by GDM status. Continuous variables were presented as means and standard deviations, while categorical variables as counts and percentages. Student t-tests were used to compare differences between group means for continuous variables and Pearson Chi-square tests were used for categorical variables. Univariate and multivariate regression models were used to quantify the association between the different sociodemographic and pregnancy-related variables and GDM status, and between GDM status and the adverse outcomes. Crude and adjusted odds ratios (aOR) with 95% confidence intervals (CI) were calculated. Statistical analyses were performed using Stata 15.1 (Stata Corp, College Station, TX). A p-value less than or equal to 0.05 defined statistical significance.

## Results

404 pregnant women with twin pregnancies were included in this study. The study population had a mean age of 30.1 (SD: 5.3) and were majority multiparous (66.6%). The average parity of each woman was about two while the average gravidity was about four. Most of the population was overweight or obese (66.5%). About 42.9% of the population had gotten pregnant with assisted reproductive technology. Amongst the known methods of conception*, In-Vitro Fertilization (IVF)* was the assistance that majority of the population used (68.8%).

About 27.0% of the women were diagnosed with GDM in their pregnancy. The characteristics of the women before and at during the pregnancy by GDM status are presented in Table 1. In general, women diagnosed with GDM were older (31.9 ± 2.4 versus 29.4 ± 5.2, *p* < 0.001), heavier at conception (74.0 ± 18.6 versus 68.2 ± 14.3, *p* = 0.008), and less likely to be nulliparous (24.7% versus 36.5%, *p* = 0.058). They were also more likely to have higher blood pressures, both systolic and diastolic. On average, a woman with GDM would have a systolic blood pressure of 117.3 mm/hg (SD: 12.7) while a woman without would have a reading of 112.1 mm/hg (SD: 11.9) (*p* = 0.001). 14.7% of women who had an incidence of GDM had previously been diagnosed with GDM in their past pregnancies while only 2.0% of those who were not diagnosed with GDM had previously been diagnosed (*p* < 0.001). Majority of women with GDM were managed with nutrition therapy (71.5%). The therapy was escalated with addition of Metformin in 11.9%, insulin in 14.7% and both insulin and metformin in 1.8% of the GDM cases.

**Table 1:** Descriptive maternal characteristics of 404 women with twin pregnancies by gestational diabetes mellitus (GDM) status in Al Ain, UAE

	**GDM**	**Non-GDM**	***p-value***
**Number (%)**	109 (27.0)	295 (73.0)	
***Age (years%)***^**ǂ**^	31.9 ± 2.4	29.4 ± 5.2	< 0.001
***Nulliparity***			
*Yes*	19 (24.7%)	80 (36.5%)	0.058
*No*	58 (75.3%)	139 (63.5%)	
***Parity***	1.95 ± 1.8	2.1 ± 1.9	0.698
***Gravidity***	4.1 ± 2.2	3.8 ± 2.1	0.358
***Weight at conception***	74.0 ± 18.6	68.2 ± 14.3	0.008
***Weight at delivery***	84.4 ± 16.0	80.8 ± 15.3	0.061
***BMI***			0.066
*Normal*	17 (24.3%)	58 (37.7%)	
*Overweight*	26 (37.1%)	57 (37.0%)	
*Obesity*	27 (28.6%)	39 (25.3%)	
***Weight gain***	9.6 ± 7.6	11.4 ± 7.0	0.089
***SBP***	117.3 ± 12.7	112.1 ± 11.9	0.001
***DBP***	72.7 ± 10.3	69.9 ± 8.5	0.021
***Mode of conception***			
*Assisted*	42 (43.30%)	108 (42.7%)	0.918
*Spontaneous*	55 (56.7%)	145 (57.3%)	
***Smoking***			
*Yes*	0 (0.00%)	1 (0.3%)	0.543
*No*	109 (100%)	294 (99.7%)	
***Previous GDM***			
*Yes*	16 (14.7%)	6 (2.0%)	< 0.001
*No*	93 (85.3%)	289 (98.0%)	
***Previous Hypertension***			
*Yes*	0(0.0%)	3 (1.0%)	0.291
*No*	109 (100%)	292 (99.0%)	

There were no differences in the mean parity, gravidity, weight gain, weight at delivery, smoking, previous hypertension, or mode of conception between women with and without GDM.

There were no significant differences in the maternal (Table 2) and neonatal (Table 3) outcomes between women with and without GDM. Although the premature rupture of membranes (PROM) and cholestasis showed differences between the two groups, they did not reach statistical significance. However, women who had GDM were hospitalized for a larger number of average days (1.4 versus 0.9 days, *p* = 0.002).

**Table 2:** Maternal outcomes in 404 women with twin pregnancies by gestational diabetes mellitus (GDM) status in Al Ain, UAE

	**GDM** (*n* = 109, 27.0%)	**Non- GDM** (*n* = 295, 73.0%)	***p-value***
***Mode of delivery***			*0.189*
*CS (either one or both babies)*	83 (76.2%)	205 (69.5%)	
*Non-CS delivery*	26 (23.8%)	90 (30.5%)	
***Premature delivery***			*0.290*
*Yes*	67 (61.5%)	164 (55.6%)	
*No*	42 (38.5%)	131 (44.4%)	
***Hypertensive disorders***			*0.701*
*Yes*	7 (6.4%)	16 (5.4%)	
*No*	102 (93.6%)	279 (94.6%)	
***PROM***			*0.057*
*Yes*	12 (11.0%)	56 (19.0%)	
*No*	97 (89.0%)	239 (81.0%)	
***Polyhydramnios***			*0.175*
*Yes*	1 (0.9%)	10 (3.4%)	
*No*	108 (99.1%)	285 (96.6%)	
***Cholestasis***			*0.065*
*Yes*	0 (0%)	9 (3.0%)	
*No*	109 (100.0%)	286 (97.0%)	
***Hospitalized days***	1.4 ± 1.3	0.9 ± 0.9	*0.002*
***Gestational age at birth***	34.8 ± 2.9	34.9 ± 3.4	*0.848*

**Table 3:** Neonatal outcomes in 404 women with twin pregnancies by gestational diabetes mellitus (GDM) status in Al Ain, UAE

	**GDM** (*n* = 109, 27.0%)	**Non- GDM** (*n* = 295, 73.0%)	***p-value***
**Small for gestational age**			*0.824*
*Yes*	59 (54.1%)	156 (52.9%)	
*No*	50 (45.9%)	139 (47.1%)	
**Large for gestational age**			*0.256*
*Yes*	8 (7.3%)	33 (11.2%)	
*No*	101 (92.7%)	262 (88.8%)	
**Macrosomia**			*0.471*
*Yes*	4 (3.7%)	16 (5.4%)	
*No*	105 (96.3%)	279 (94.6%)	
**APGAR 1 min < 7**			*0.368*
*Neither baby*	78 (82.1%)	209 (86.0%)	
*One or both twins*	17 (17.9%)	34 (14.0%)	
**APGAR 5 min < 7**			*0.767*
*Neither baby*	98 (97.0%)	268 (96.4%)	
*One or both twins*	3 (3.0%)	10 (3.6%)	
**Major malformations**			*0.82*
*Neither baby*	99 (90.8%)	270 (91.5%)	
*At least one baby*	10 (9.2%)	25 (8.5%)	
**Death**			*0.633*
*Neither baby*	101 (93.5%)	271 (94.8%)	
*At least one baby*	7 (6.5%)	15 (5.2%)	
**RDS**			*0.686*
*Neither baby*	60 (55.1%)	169 (57.3%)	
*At least one baby*	49 (44.9%)	126 (42.7%)	
**Hemorrhage**			*0.140*
*Neither baby*	108 (99.1%)	284 (96.3%)	
*At least one baby*	1 (0.9%)	11 (3.7%)	
**Sepsis**			*0.612*
*Neither baby*	97 (89.0%)	257 (87.1%)	
*At least one baby*	12 (11.0%)	38 (12.9%)	
**Retinopathy**			*0.227*
*Neither baby*	105 (99.1%)	282 (96.9%)	
*At least one baby*	1 (0.9%)	9 (3.1%)	
**Jaundice**			*0.777*
*Neither baby*	50 (45.9%)	140 (47.5%)	
*At least one baby*	59 (54.1%)	155 (52.5%)	
**NICU**			*0.343*
*Neither baby*	50 (45.9%)	151 (51.2%)	
*At least one baby*	59 (54.1%)	144 (48.8%)	
**Hypoglycemia**			*0.380*
*Neither baby*	102 (93.6%)	268 (90.9%)	
*At least one baby*	7 (6.4%)	27 (9.2%)	

***RDS respiratory distress syndrome, NICU admission to neonatal intensive care unit***

Table 4 shows the association between maternal factors and GDM. Age (OR 1.09, 95% CI: 1.05–1.14) and weight (1.02 95% CI: 1.01–1.04) at conception were associated with the development of GDM. Women with previous pregnancies which were exposed to GDM were 8.29 times more likely to have GDM in this index twin pregnancy in the crude analyses. There was also a slight increase in odds of being diagnosed with GDM if the woman’s SBP and DBP at conception were higher (OR: 1.01, 95% CI: 1.00–1.01 for both blood pressures). Furthermore, upon adjusting for age, both weight and past GDM continued to be associated to the incidence of GDM with adjusted odds ratios (aOR) of 1.02 (95% CI: 1.00–1.04) and 7.37 (95% CI: 2.76–19.73), respectively.

**Table 4:** Associations between maternal factors and the incidence of gestational diabetes mellitus in women with twin pregnancies in Al Ain, UAE

	**Unadjusted odds ratio (95% CI)**	**Age-adjusted odds ratio** **(95% CI)**
*Weight at conception*	**1.02 (1.01–1.04)**	**1.02 (1.00–1.04)**
*Age*	**1.09 (1.05–1.14)**	
*Gravidity*	1.06 (0.94–1.19)	0.94 (0.82–1.08)
*Nulliparity*	0.57 (0.32–1.02)	0.76 (0.41–1.41)
*Past GDM*	**8.29 (3.15–21.80)**	**7.37 (2.76–19.73)**
*SBP at conception*	**1.01 (1.00–1.01)**	**1.04 (1.01–1.06)**
*DBP at conception*	**1.01 (1.00–1.01)**	**1.03 (1.00–1.06)**
*Assisted mode of conception*	1.03 (0.64–1.64)	1.02 (0.63–1.65)

The crude and adjusted association between GDM and maternal and neonatal outcomes are presented in Table 5. GDM was associated with hospitalization during twin pregnancy and the advent of a cesarean section in both the crude and adjusted models. In regression model adjusted for age, GDM was associated with a 1.60 times (95% CI: 1.16–2.20) increased odds of being hospitalized. On the other hand, after being adjusted for age, BMI at conception and gravidity, GDM was associated with 2.34 times (95% CI: 1.03–5.30) increased odds of having cesarean section (Table 5). No significant associations were detected between GDM in twin pregnancy and odds of small for gestational age, macrosomia, premature birth or hypoglycemia.

**Table 5:** Associations between gestational diabetes mellitus and maternal and neonatal outcomes in women with twin pregnancies in Al Ain, UAE

	**Unadjusted odds ratio (95% CI)**	**Adjusted**^*a*^ **odds ratio (95% CI)**
*Hospitalization during pregnancy*^*b*^	**1.67 (1.21 -2.81)**	**1.60 (1.16–2.20)**
*Cesarean Section*	1.25 (0.84–1.84)	**2.34 (1.03–5.30)**
*PROM*	0.53 (0.27–1.03)	0.31 (0.08–1.19)
*Small for gestational age*	1.05 (0.68–1.63)	1.45 (0.73–2.88)
*Macrosomia*	0.66 (0.22–2.03)	0.64 (0.11–3.58)
*Premature birth*	1.30 (0.82–2.06)	1.65 (0.83–3.29)
*Hypoglycemia*	0.68 (0.29–1.61)	0.91 (0.21–3.89)

^*a*^*Models adjusted for age, body mass index and gravidity*

^*b*^*Model adjusted for age only*

## Discussion

The incidence of GDM in this population was about 27.0%. Age and weight at conception were significantly associated with the development of GDM in twin pregnancies, while GDM was significantly associated with hospitalization during twin pregnancy and the advent of a cesarean section in these pregnancies.

Twin pregnancies have an increased risk of GDM as this study shows. The rates of GDM in this twin pregnancies population is higher than other singleton pregnancy populations in the region and around the world. This might presumably indicate that twin pregnancies are likely to cause increased incidence of GDM than singleton pregnancies. This could be due to several reasons such as the increased weight gain in twin pregnancies, the use of assisted technology to achieve a multiple pregnancy and increased blood pressures before and during the pregnancy.

The effect of age and weight on GDM on both twin and singleton pregnancies have been extensively studied [32]. Older and overweight and obese women with twin pregnancies are more at risk of being diagnosed with GDM even after adjustment of multiple other covariates [33]. Moreover, women with higher blood pressure profiles at conception have been shown to be at risk of GDM as well as pre-eclampsia in twin pregnancies [34].

For women with type 1 and 2 diabetes mellitus and GDM, poor maternal glycemic control can significantly increase maternal and neonatal risk for adverse outcomes. Nutritional and pharmaceutical therapies are usually recommended for women with diabetes to ensure proper glycemic control during pregnancy. Despite this, women with diabetes often require inpatient diabetes management or hospitalizations before their delivery to ensure that maternal hyperglycemia does not significantly increase the risk of adverse outcomes for the mother and child [35]. This seems to be the case in this population that had higher rates of hospitalization for women with GDM. Hence twin pregnancy with GDM is considered high-risk pregnancy, obstetricians at our institutions have very low threshold for admission for different complaints, Moreover, it is also common practice among obstetricians to admit patients for glycemic control, as many patients prefer admission than frequent outpatient visits.

Different clinical characteristics and poorer pregnancy outcomes were found among GDM twin pregnancies when compared with non-GDM twin pregnancies and GDM singleton pregnancies, including risk of new onset gestational hypertension, preeclampsia, neonatal ICU admission, prematurity and perinatal mortality [36]. Cesarean section was independently associated with GDM in twin pregnancies in our population. While cesarean section is already prevalent in twin pregnancies (71% in our population), it seemed to have an increased association when attributed to those with GDM. Although we were unable to delve deeper into this matter, a systematic review on the cesarean section associations with twin pregnancies has shown that cesarean section is common in twin pregnancies due to growth retardation of a twin in the pregnancy [37]. It is also prescribed during delivery when one of the twins goes into breech.

Several studies have shown that despite GDM being a predictor of macrosomia in singleton pregnancies, it is not the case in twin pregnancies [38]. This is supported by our findings as no significant association was detected. In twin pregnancies, it has been previously shown that GDM, when well monitored either via therapy or diet, has an opposite effect to the size of the baby. As such, there is sometimes an effect between twin pregnancies, GDM and small for gestational ages. However, we did not find this association in our study. Similar to results from a meta-analysis on GDM in twin pregnancies, our study found no associations to birth weight, gestational age, large or small for gestational age neonates, respiratory distress, neonatal hypoglycemic or low Apgar score [39].

Although, both singleton and twin GDM pregnancies are high-risk pregnancies which require optimal management, currently, there is little evidence to guide the management of GDM twin pregnancies. The optimal glucose targets, dietary requirements, and timing of delivery are uncertain, and further studies are needed to define the best management for these women. Women suffering from GDM are also more likely to suffer from an onset of Type 2 diabetes. As the rate of GDM in our population of twin pregnancies was high, it is necessary to manage these women well. Nevertheless, future research is needed to investigate the postpartum OGTT, HbaA1c levels and rates of postpartum dysglycaemia in women with twin pregnancies, and whether there is a risk difference in developing type 2 diabetes later in life between twin and singleton GDM pregnancies.

To our knowledge, this is the first study in the country to determine the incidence of GDM in twin pregnancies. Although the study could not confirm significant associations between GDM and neonatal outcomes, it was in line with most literature depicting no significant increase in neonatal outcomes due to the GDM and twin pregnancy [39].

Our study included only women with twin pregnancies, and therefore direct comparisons to the prevalence and adverse outcomes of GDM amongst singleton pregnancies were not possible. We were also unable to determine if the cesarean section were of elective or inductive in nature, as during the study period, there was no specific protocol for managing the delivery of twin pregnancies in both institutions. However, the NICE guideline management of multiple pregnancies endorsed by Royal College of Obstetricians was followed mostly and therefore timing of birth for women with uncomplicated monochorionic twin pregnancies was predetermined between 36–37 weeks of gestational age and for women with uncomplicated dichorionic twin pregnancies between 37–38 weeks.

Although BMI as an adjustment would have been a better quality covariate, we had a high level of missing height measurements in the model. However, weight has been used instead and has shown to be used in similar populations with similar research queries [33]. Although this retrospective chart review was only done in two major hospitals in Al Ain, the generalizability of the data to the Emirati population is probable. All of the Emirati population have full health insurance coverage providing them with the same level of health care at any health facility. As such, there is no difference in healthcare access between pregnant women attending these two hospitals and those who use other institutions. Therefore, a representative sample of the Emirati population in Al Ain can be recruited from these two hospitals.

## Conclusion

In the present study, more than a quarter of women with twin pregnancies were diagnosed with GDM and associated with some adverse pregnancy outcomes but not fetal outcomes. The study findings support the need to determine interventions that would reduce hospitalization and delivering via cesarean section for women who suffer from GDM in their twin pregnancies. More studies are need to further investigate the management of GDM in twin pregnancies and its effects and associations with adverse outcomes.

## Data Availability

The datasets used and/or analyzed during the current study are available from the corresponding author on reasonable request.

## Abbreviations

GDM: Gestational diabetes mellitus
UAE: United Arab Emirates
CI: Confidence intervals
